# Nrf2 Activation and Its Coordination with the Protective Defense Systems in Response to Electrophilic Stress

**DOI:** 10.3390/ijms21020545

**Published:** 2020-01-15

**Authors:** Takamitsu Unoki, Masahiro Akiyama, Yoshito Kumagai

**Affiliations:** 1 Department of Basic Medical Sciences, National Institute for Minamata Disease, Minamata, Kumamoto 867-0008, Japan; 2 Environmental Biology Laboratory, Faculty of Medicine, University of Tsukuba, Tsukuba, Ibaraki 305-8575, Japan; akiyama.masahiro.ga@u.tsukuba.ac.jp (M.A.); yk-em-tu@md.tsukuba.ac.jp (Y.K.)

**Keywords:** electrophiles, covalent modification, NF-E2-related factor 2, Kelch-like ECH-associated protein 1, reactive sulfur species, cystathionine γ-lyase

## Abstract

Molecular responses mediated by sensor proteins are important for biological defense against electrophilic stresses, such as xenobiotic electrophile exposure. NF-E2-related factor 2 (Nrf2) has an essential function as a master regulator of such cytoprotective molecular responses along with sensor protein Kelch-like ECH-associated protein 1. This review focuses on Nrf2 activation and its involvement with the protective defense systems under electrophilic stresses integrated with our recent findings that reactive sulfur species (RSS) mediate detoxification of electrophiles. The Nrf2 pathway does not function redundantly with the RSS-generating cystathionine γ-lyase pathway, and vice versa.

## 1. Introduction

Humans are exposed to various xenobiotic electrophiles on a daily basis through their dietary habits, living environment, lifestyle and so on. Examples of xenobiotic electrophiles include methylmercury (MeHg) in fish [1], cadmium (Cd) in rice [2], and 1,2-napthoquinone (1,2-NQ) and 1,4-napthoquinone (1,4-NQ) in the atmosphere [3]. Electrophiles are electron-deficient species that form covalent bonds with electron-rich nucleophiles. Nucleophilic biomacromolecules, such as the side chains on protein amino acids (e.g., Cys, His, Arg and Lys) and aromatic nitrogen sites on DNA bases, are targeted by electrophiles to form stable adducts [4]; thus, these electrophile–nucleophile interactions are involved in deleterious effects [5,6,7,8,9]. However, the chemical modification of sensor proteins with reactive thiols by xenobiotic electrophiles at low doses results in the activation of cellular signal transduction pathways to maintain cellular homeostasis [5,6,8,10,11,12]. This adaptive cellular response to electrophiles is termed electrophilic signaling and involves both basal metabolism and oxidative/inflammatory responses [13,14,15,16]. Upon such electrophilic stresses, electrophilic modifications of sensor protein Kelch-like ECH-associated protein 1 (Keap1) activates transcription factor NF-E2-related factor 2 (Nrf2), a master regulator of cytoprotective molecules.

Reactive sulfur species (RSS), sulfane sulfur-containing highly nucleophilic molecules, plays a role in protection against electrophilic stresses through sulfur adduct formation of electrophiles [17]. The trans-sulfuration enzyme cystathionine γ-lyase (CSE) catalyzes the production of cysteine persulfide (CysSSH) from cystine (CysSSCys) as a substrate [18]. In turn, CysSSH spontaneously produces other reactive persulfide/polysulfide species, such as GSSH [18,19]. Our recent study clarified the contributions of Nrf2 and CSE to the blockage of electrophilic stresses in a parallel mode [20].

## 2. Nrf2 Activation under Electrophilic Stress

Nrf2 is a key regulator of cellular defenses against oxidative, electrophilic and environmental stresses. In the nucleus, Nrf2 forms a dimer with the small Maf protein (sMaf) and binds to the CNC-sMaf binding element (CsMBE) (formerly described as the antioxidant/electrophile response element (ARE/EpRE)) located in the regulatory regions of many genes responsible for cellular defense [21,22]. Nrf2 cooperatively regulates antioxidant proteins; such as heme oxygenase-1 (HO-1) and glutamate cysteine ligase (GCL), to synthesize glutathione (GSH); phase-II xenobiotic detoxifying enzymes, such as GSH *S*-transferase (GST) and UDP-glucuronosyltransferase (UGT); and phase-III xenobiotic transporters, such as multidrug resistance-associated protein (MRP) [21,23,24,25,26,27,28]. GSH is an abundant sulfhydryl found in various tissues that plays an important role in detoxification of electrophiles via GSH adduct formation. Although the p*K*a value of GSH is relatively high (9.12), indicating that about 2% of GSH exists as GS^-^, its deprotonated form that interacts with electrophiles, GST is able to lower this p*K*a value, thereby facilitating GSH adduct formation with electrophiles [29,30]. Such a polar metabolite is associated with detoxification of electrophiles because the GSH adduct is then excreted into the extracellular space through MRP [31]. These findings suggest that Nrf2 is essential for repression of environmental electrophile-mediated toxicity through GSH adduct formation and its excretion into the extracellular space.

**Figure 1:**
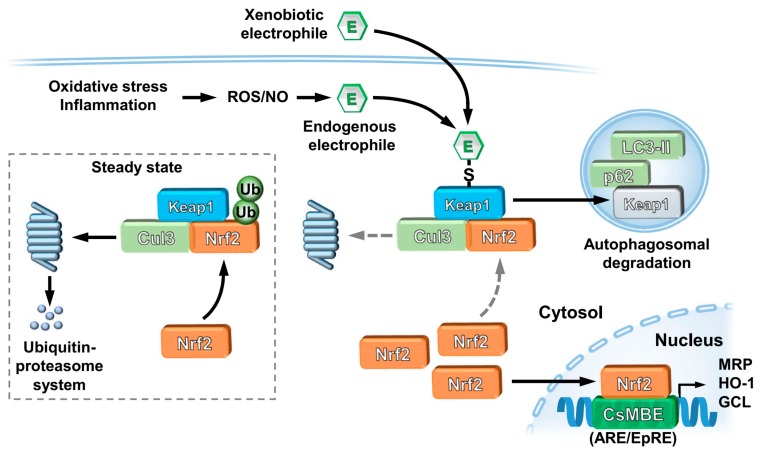
Nrf2 activation mechanisms under electrophilic stresses. Under steady state conditions, Nrf2 is captured by Keap1 and constantly ubiquitinated by Cul3 in the cytosol leading to degradation through the ubiquitin–proteasome system and resulting in the inhibition of Nrf2 translocation from the cytoplasm to the nucleus (inset). Keap1 is modified through its highly reactive cysteine residues by electrophiles that are exposed exogenously or generated endogenously as second messengers, thereby diminishing its activity to hold Nrf2. As a result, Nrf2 newly synthesized translocates into the nucleus and interacts with a partner protein sMaf resulting in formation of a heterodimer that binds to the CsMBE (formerly known as ARE or EpRE), thereby upregulating its downstream genes that are involved in cellular defense (e.g., MRP, HO-1 and GCL). Autophagosomal degradation of Keap1 is also upregulated through its electrophilic modifications. Dotted gray arrows indicate processes disrupted by electrophilic stresses.

Nrf2 is regulated by Kelch-like ECH-associated protein 1 (Keap1), an adaptor subunit of Cullin 3-based E3 ubiquitin ligase (Cul3). Under normal conditions, Keap1 binds to Nrf2 in the cytoplasm and promotes the ubiquitination and proteasomal degradation of Nrf2 and, thus, acts as a negative regulator of Nrf2 [32,33,34]. Keap1 is also a sensor protein for oxidative and electrophilic insults through the modification of its highly reactive cysteine residues. Thus, when the interaction between Nrf2 and Keap1 is disrupted, proteasomal degradation of Nrf2 decreases, causing de novo Nrf2 to build up within the cell, leading to increased translocation of Nrf2 into the nucleus (Figure 1).

### 2.1. Covalent Modification of Keap1

**Figure 2:**
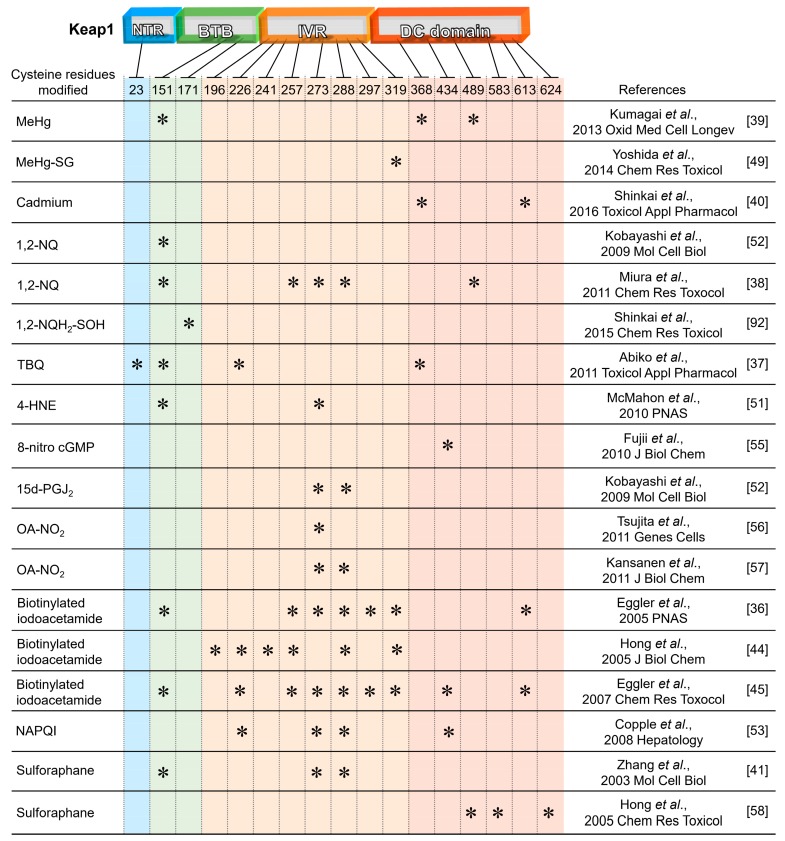
The cysteine code of Keap1 modification sites with various electrophiles. The BTB (Board complex, Tramtrack and Bric-à-brac), IVR (intervening region) and DC (DGR/CTR: double glycine repeat/C-terminal region) domains are essential for the Keap1 homodimerization, heterodimerization with Cul3 and the binding of Nrf2, respectively. ⁎, cysteine residue(s) modified by each electrophile listed.

Keap1 modification sites, consisting of the NTR (N-terminal region), BTB (Board complex, Tramtrack and Bric-à-brac), IVR (intervening region) and DC (DGR/CTR: double glycine repeat/C-terminal region) domains, can be modified by a variety of electrophiles [35,36,37,38,39,40]. Among them, Cys151, Cys273 and Cys288 are established as being essential for regulating Nrf2 function [35,41,42,43]. Electrophile-mediated Keap1 modification sites are shown in Figure 2. Studies with biotinylated iodoacetamide, a thiol blocking agent, revealed diverse Keap1 modification sites [36,44,45]. MeHg binds to recombinant Keap1 protein, and results from a biotin-PEAC_5_-maleimide labeling assay also revealed that Keap-1 is a target for *S*-mercuration in SH-SY5Y cells [46,47]. Further studies indicated that MeHg can specifically bind to three cysteine residues in Keap1, Cys151, Cys368 and Cys489, and induce Nrf2 activation and subsequent upregulation of the GCL catalytic subunit (GCLC) and GCL modifier subunit (GCLM) in SH-SY5Y cells [39]. In the intracellular space, MeHg is readily conjugated to GSH to form a MeHg-SG adduct that is transferred to the extracellular space through MRP [48]. In this sense, using a synthetic ethyl monoester of MeHg-SG, Yoshida et al. showed that this adduct induced a concentration-dependent toxicity and activate Nrf2-related genes in SH-SY5Y cells. This is because the Hg-S bond in MeHg-SG is relatively unstable and thus this GSH adduct readily undergoes *S*-transmercuration with cellular proteins, including Keap-1, forming protein–MeHg adducts [49]. While MeHg modifies the Cys151 residue in Keap-1, the MeHg-SG adduct modifies the Cys319 residue. This residue is located in the IVR region of Keap1 and is important to ubiquitin E3 ligase activity and Nrf2 degradation [50]. Thus, it is possible to speculate that the *S*-mercuration in the Cys319 residue also contributes to the up-regulation of Nrf2 in response to MeHg [47]. Shinkai et al. showed that Cd modifies recombinant Keap1 protein at Cys249, Cys368 and Cys613 [40]. Furthermore, McMahon et al. reported that His225, Cys226 and Cys613 in Keap1 are functionally involved in the Cd-induced activation of Nrf2 by using a mutated form of Keap1 [51]. Taken together, Cys613 seems to play a critical role in the activation of the Keap1–Nrf2 system by Cd. In bovine vascular endothelial cells, Keap1-mediated Nrf2 activation is involved in Cd-induced metallothionein (MT) expression in addition to HO-1, GCLM and NAD(P)H quinone dehydrogenase 1 (NQO1) expression [40]. 1,2-NQ modified recombinant Keap1 protein at Cys151, Cys257, Cys273, Cys288 and Cys489, and upregulated GCLC, GCLM, GST alpha (GSTA), GST mu (GSTM), UGT1A and NQO1 in primary mouse hepatocytes [38,52]. Another type of quinone, *tert*-butyl-1,4-benzoquinone (TBQ), an electrophilic metabolite of hydroxyanisole, modified Keap1 through Cys23, Cys151, Cys226 and Cys368, and upregulated GCLC, GCLM, GSTA, NQO1 and HO-1 in RAW264.7 cells [37]. Deletion of Nrf2 has been shown to enhance the cellular toxicity of these compounds, confirming that the Keap1-Nrf2 system is essential for the repression of electrophilic stresses induced by xenobiotic electrophiles. N-Acetyl-p-benzoquinone (NAPQI), which is an electrophilic metabolite of acetaminophen, modified Keap1 through Cys226, Cys273, Cys288 and Cys434, and upregulated GCLC in Hepa-1c1c7 cells [53].

The cysteine code is an attractive point to understand the nature of Keap1 function as the sensor for electrophilic compounds. But the sensing mechanism is complicated. Saito et al. analyzed the specificity of Keap1 cysteine residues against chemical inducers of Nrf2 using mutants of three major cysteine residues, Cys 151, Cys273 and Cys288 of Keap1 [54]. They classified Nrf2 inducers into four classes: class I (Cys151 preferring), class II (Cys288 preferring), class III (Cys151/Cys273/Cys288 collaboration preferring) and class IV (Cys151/Cys273/Cys288 independent). However, it seems difficult to find the chemical consistency of each class. For example, both 15-deoxy-prostaglandin J_2_ (15d-PGJ_2_) and prostaglandin A_2_ (PGA_2_) belong to the prostaglandin family and share similar structural characteristics; 15d-PGJ_2_ and PGA_2_ belong to class II and class IV, respectively. Diethyl maleate and 1-[2-cyano-3,12-dioxooleana-1,9(11)-dien-28-oyl]imidazole, which differ greatly in structure and size, both belong to class I. Therefore, the complex properties of electrophiles may be involved in the determination of the Keap1 modification site(s). Keap1 may sense various electrophilic substances through the combination of various cysteine residues.

Short-lived reactive oxygen species (ROS) and nitrogen oxide (NO) react with cyclic nucleotides and fatty acids to generate 8-nitroguanosine 3’,5’-cyclic monophosphate (8-nitro-cGMP) and nitro-oleic acid (OA-NO_2_), respectively, as endogenous electrophiles [15,16]. These electrophilic second messengers are able to modify Keap1, leading to Nrf2 activation of anti-oxidative and anti-inflammatory genes [55,56,57]. Several biologically relevant electrophiles, such as 15deoxy-Δ^12,14^-prostaglandin J_2_ (15d-PGJ_2_), sulforaphane and 4-hydroxynonenal (4-HNE), covalently react with Keap1 [41,51,52,58,59]. Modification of Keap1 cysteine residue(s) by *S*-guanylation through 8-nitro-cGMP and by Michael addition through PGJ_2_ and 4-HNE is irreversible [42,60], whereas that of OA-NO_2_ and sulforaphane is reversible [57,61].

### 2.2. Autophagosomal Degradation of Keap1

Analysis of liver-specific Atg7 (autophagy related 7)-deficient mice revealed that p62 protein is abnormally accumulated and Nrf2 is constitutively activated when the function of autophagy is disrupted. In this case the Keap1 protein is upregulated, suggesting that the Keap1 protein is constitutively degraded through the autophagy pathway [62]. Treatment of cells with TBQ, diethyl maleate (DEM) or 1,2-NQ accelerated the Keap1 degradation, indicating that Nrf2 accumulation is the dominant cause to provoke liver damage in autophagy-deficient mice. The autophagy pathway maintains the integrity of the Keap1–Nrf2 system for normal liver function by governing Keap1 turnover [63] (Figure 1).

### 2.3. Keap1-Independent Pathway

Recent findings have also revealed that Keap1-independent signal pathways might contribute to electrophile-induced Nrf2 activation and cytoprotective responses against electrophile exposure. Culbreth et al. found a Keap1-independent Nrf2 activation pathway in MeHg-exposed rat primary astrocytes, possibly involving the regulation of a member of the src family kinase named Fyn [64,65]. It has been reported that Fyn can phosphorylate Nrf2 favoring its export from the cell nucleus [66,67]. MeHg exposure in vitro increased gene expression of HO-1 and NQO1 as well as nuclear Nrf2 localization.

Oncogenic signal pathways are involved in Nrf2 expression at the transcription level. Oncogenic alleles of KRAS (kirsten rat sarcoma viral oncogene homolog), BRAF (v-raf murine sarcoma viral oncogene homolog B1) and cMYC (avian myelocytomatosis virus oncogene cellular homolog) increase Nrf2 mRNA level through binding to TPA response element located in the Nrf2 promoter region [68,69,70]. Such an oncogene-induced Nrf2 transcription upregulates antioxidant and drug-resistant capacity in tumorigenic cells. In this sense, the application of Nrf2 inhibitors to anti-cancer treatment is of interest [68].

## 3. The Nrf2 and CSE Pathways: Parallel Contribution to the Repression of Electrophilic Stress

Reactive sulfur species (RSS) containing mobilized sulfur atom(s) bound to the thiol groups of CysSH and GSH or hydrogen sulfide (H_2_S) (e.g., CysSSH/CysSSSH, GSSH/GSSSH and HSSH/HSSSH) have been shown to be highly nucleophilic and antioxidative molecules [13,18,71]. The p*K*a value of RSS is lower than that of monosulfides, such as GSH and CysSH; e.g., CysSSH has a p*K*a value of 4.34 as predicted by chemical calculations [72]. This implies that RSS predominantly exists as the deprotonated form of SH (thiolate ion, S^-^) at physiological pH. Further, RSS such as GSSH and CysSSH, but not monosulfides such as GSH and CysSH, contain mobilized sulfur capable of capturing electrophiles. This leads to the formation of sulfur adducts [19,73,74].

### 3.1. Protective Function of RSS against Electrophilic Stress

As noted above, electrophiles can be captured by RSS, leading to the formation of their sulfur adducts. For example, we previously reported that MeHg, Cd, 1,4-NQ and NAPQI, which is the toxic metabolite of acetaminophen, can react with RSS, such as H_2_S, CysSSH, GSSH, GSSSG or the model polysulfide Na_2_S_4_, leading to the formation of bismethylmercury sulfide [(MeHg)_2_S], cadmium sulfide (CdS), CdS_2_O_3_, 1,4-NQ-SH, 1,4-NQ-S-1,4-NQ, 1,4-NQ-S-1,4-NQ-OH, NAPQIH_2_-SSSCys, NAPQIH_2_-SSCys and NAPQIH_2_-SSG [5,12,75,76,77,78]. Additionally, (MeHg)_2_S and NAPQIH_2_-SSSCys were identified as novel metabolites of MeHg and acetaminophen from the liver and urine of mice treated with these compounds [78,79]. Remarkably, sulfur adduct electrophiles such as (MeHg)_2_S, CdS and 1,4-NQ-S-1,4-NQ-OH are less electrophilic and toxic than their parent compounds; thus, they are defined as detoxified metabolites [75,76,77]. This indicates that RSS are involved in the repression of electrophile-mediated toxicity by the formation of inactive metabolites of electrophiles. Consistent with this notion, the toxicity of MeHg, Cd and 1,4-NQ was effectively blocked by treatment with the model polysulfide Na_2_S_4_ [75,77,80]. 

**Figure 3:**
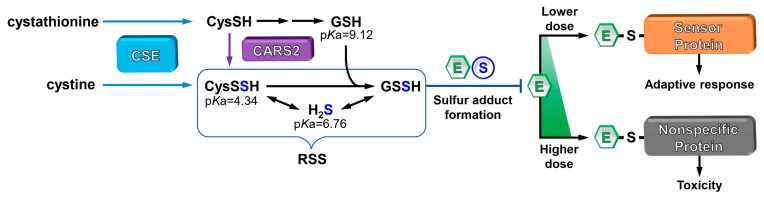
RSS generation and its negative regulation of covalent modification of cellular proteins by xenobiotic electrophiles. CSE- and CARS2-mediated metabolisms are shown as the blue arrows and the purple arrow, respectively. The xenobiotic electrophile-mediated activation (low dose) and disruption (high dose) of redox signaling by sensor proteins with low p*K*a thiols and effector molecules are negatively regulated by RSS through sulfur adduct formation. E, xenobiotic electrophiles; RSS, reactive sulfur species.

While cystathionine γ-lyase (CSE) has been known as the final trans-sulfuration enzyme essential for cysteine biosynthesis from cystathionine [81], we reported that CSE can also catalyze the production of cysteine persulfide (CysSSH) from cystine (CysSSCys) as a substrate [18] (Figure 3). In turn, CysSSH spontaneously produces other reactive persulfide/polysulfide species, such as GSSH, and also H_2_S as a derivative [18,19]; thus, CSE act as an RSS generating enzyme (Figure 3). Mitochondrial cysteine-tRNA synthetase 2 (CARS2) also catalyzes formation of CysSSH from CysSH [82] (Figure 3). The metabolite analysis of wild-type (WT) and CSE knockout (KO) mice exposed to MeHg showed that (MeHg)_2_S was present at significant levels in various WT mouse tissues, but not at appreciable levels in CSE KO mouse tissues [79], suggesting that CSE is a critical enzyme involved in capturing xenobiotic electrophiles through formation of sulfur adducts in vivo. Furthermore, knockdown of CSE enhanced Cd-induced cytotoxicity, whereas overexpression of CSE protected cells from this electrophile-induced cytotoxicity [83]. In vivo studies have shown that CSE deletion in mice causes increased sensitivity to acetaminophen-induced hepatotoxicity, Cd-induced hepatotoxicity and MeHg-induced toxicity [20,77,84]. Overall, this suggests that CSE is a crucial factor engaged in the protection against electrophiles because this enzyme is involved in the production of RSS, thereby capturing electrophiles to yield their sulfur adducts [5,85] (Figure 3). Xenobiotic electrophiles selectively modify sensor proteins through thiolate ions at low doses and thus inhibit their activities. As a result, activation of effector molecules leads to a cellular adaptive response. However, a higher dose of xenobiotic electrophiles causes nonspecific protein modification, disrupting these signals, leading to cell death. Our previous studies confirmed such a dose-dependent transition, focusing on some sensor proteins that mediate cellular redox signaling pathways during exposure to xenobiotic electrophiles; phosphatase and tensin homologue (PTEN) modification by MeHg [11] and 1,4-NQ [75] as well as heat shock protein 90 (HSP90) by 1,4-NQ [12] and cadmium [77]. The fact that these xenobiotic electrophile-mediated redox signaling pathways and cellular toxicity are negatively regulated by RSS through the formation of their sulfur adducts, indicate that RSS act as the initial defense system for xenobiotic electrophile exposure [12,75,77] (Figure 3).

### 3.2. The Nrf2 and CSE Pathways Contribute to the Elimination of Electrophilic Stress in a Parallel Manner

Nrf2 plays a key role in the detoxification of electrophiles via formation of GSH adducts and subsequent excretion into extracellular spaces, whereas the CSE-mediated defense mechanism is facilitated through the sulfur adduct formation by RSS. These findings suggest that there is a non-canonical pathway of detoxification of environmental electrophiles conducted by CSE in addition to the canonical pathway regulated by Nrf2. In fact, CSE/Nrf2 double-KO mice and their hepatocytes were more sensitive to various electrophiles, such as MeHg, Cd, 1,4-NQ, crotonaldehyde and acrylamide, than their single-KO counterparts [20], indicating that both factors are involved in the repression of electrophile-induced toxicity in parallel pathways. Thus, not only does GSH adduct formation regulated by Nrf2 play a critical role in the protection against electrophiles but so does sulfur adduct formation regulated by CSE-producing RSS. Collectively, critical and parallel participation of Nrf2 and CSE in the protection against environmental electrophiles is like two sides of the same coin.

Incidentally, our study also revealed that CSE deletion did not substantially affect the cytotoxicity elicited by MeHg, 1,4-NQ, crotonaldehyde and acrylamide in primary mouse hepatocytes, in contrast to Nrf2 deletion [20]. A possible cause of this observation is that alternative defense pathway(s) may compensate for the lack of CSE and protect cells from the electrophilic damage. We found that CSE deletion enhanced Cd-mediated activation of Nrf2 and its downstream protein HO-1 in primary mouse hepatocytes [20]. Such a phenomenon may be also seen in MeHg and 1,4-NQ because of exposure of cultured cells to MeHg [46,65,86] and the 1,4-NQ-activated [87] Nrf2 signaling pathway. These findings suggest that the function of Nrf2 is potentiated in CSE deficient hepatocytes, thus protecting cells from xenobiotic electrophiles.

## 4. Discussion

**Figure 4:**
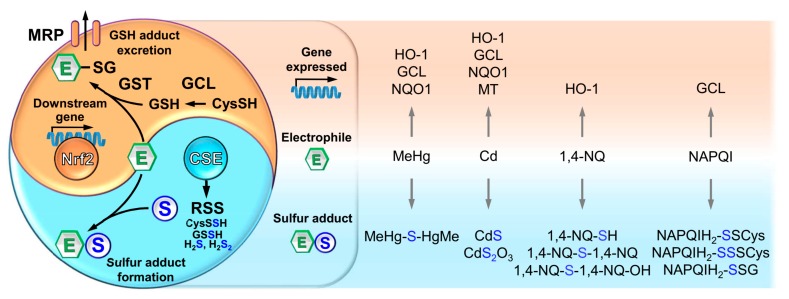
The Nrf2 and CSE pathways contribute to the elimination of electrophilic stress in a parallel manner. Nrf2 is a central coordinator for the induction of detoxifying genes that in turn are translated into proteins that are associated with GSH adduct formation and subsequent excretion into the extracellular space (e.g., GCL, GST and MRP). Aside from Nrf2, RSS generation mediated by CSE contributes to detoxifying electrophiles through sulfur adduct formation. For various electrophiles, upregulated genes and sulfur adducts have been uniquely identified (gray arrows).

Figure 4 shows the integration of our knowledge of the Nrf2 and CSE pathways. Upon electrophilic stresses, numerous studies confirmed that Nrf2 acts as a master regulator for cytoprotective molecules along with its activation mechanism through Keap1 as a sensor protein. CSE is another key factor implicated in the protection against electrophilic stresses. CSE is involved in RSS production, and works to capture electrophiles to yield their sulfur adducts. RSS-mediated protection against electrophiles appears to be a primary defense system compared with the Nrf2-dependent detoxification system with GSH because RSS are constitutively produced by CSE, whereas the gene expression of GCL, GST and MRP associated with conjugation with GSH and subsequent excretion of GSH adducts into the extracellular space are cooperatively regulated by Nrf2 [21,23]. As discussed above, the Nrf2 and CSE pathways do not function redundantly because CSE/Nrf2 double-KO mice and their hepatocytes were more sensitive to various electrophiles than their single-KO counterparts [20], though it is reported that the expression of CSE is, at least in part, transcriptionally regulated by ATF4 (activating transcription factor-4) [88,89]. Some studies suggested that Nrf2 is a positive regulator of ATF4 under the condition of electrophilic stress [90,91]. Thus, cross-talk between Nrf2 signaling and CSE expression might be mediated by ATF4 in response to electrophilic stresses. It is reported that RSS/ROS mediates 1,2-NQH_2_-SOH generation as sulfenic acid from 1,2-NQ, and 1,2-NQH_2_-SOH modifies Keap1 through Cys171, thereby activating Nrf2 [92]. Thus, RSS are able to affect Nrf2 activation through electrophile metabolic fate, and further studies are required to clarify the crosstalk between the Nrf2 and CSE pathways.

There are numerous examples of the beneficial effects elicited by pretreatment with Nrf2 activators as potential preventive therapeutics [93]. In fact, isothiocyanate and sulforaphane activated Nrf2 and upregulated downstream proteins associated with MeHg excretion, and suppressed mercury accumulation and intoxication in mice exposed to MeHg [94]. We speculate that directly trapping electrophiles by RSS would also be an effective strategy for electrophile detoxification. Therefore, the intake of foods and/or supplements containing not only Nrf2 activators but also sulfane sulfur-dependent chemicals may help to diminish the health risks of electrophilic stresses. 

## Abbreviations

(MeHg)_2_S	Bismethylmercury sulfide
1,2-NQ	1,2-napthoquinone
1,4-NQ	1,4-napthoquinone
15d-PGJ_2_	15deoxy-Δ^12,14^-prostaglandin J_2_
4-HNE	4-hydroxynonenal
8-nitro-cGMP	8-nitroguanosine 3’,5’-cyclic monophosphate
ARE	Antioxidant response element
ATF4	Activating transcription factor-4
BTB	Board complex, Tramtrack, and Bric-à-brac
Cd	Cadmium
CdS	Cadmium sulfide
CSE	Cystathionine γ-lyase
CsMBE	CNC-sMaf binding element
Cul3	Cullin 3-based E3 ubiquitin ligase
DC	DGR/CTR: double glycine repeat/C-terminal region
DEM	Diethyl maleate
EpRE	Electrophile response element
GCL	Glutamate cysteine ligase
GSH	Glutathione
GST	GSH *S*-transferase
H_2_S	Hydrogen sulfide
HO-1	Heme oxygenase-1
IVR	Intervening region
Keap1	Kelch-like ECH-associated protein 1
KO	Knockout
MeHg	Methylmercury
MRP	Multidrug resistance-associated protein
MT	Metallothionein
NAPQI	N-acetyl-p-benzoquinone
NO	Nitrogen oxide
Nrf2	NF-E2-related factor 2
NTR	N-terminal region
OA-NO_2_	Nitro-oleic acid
ROS	Reactive oxygen species
RSS	Reactive sulfur species
sMaf	Small Maf
TBQ	*Tert*-butyl-1,4-benzoquinone
UGT	UDP-glucuronosyltransferase
